# Complete genome sequence of *Gulosibacter* sp. ACHW.36C, a toluene-degrading strain isolated in Hong Kong

**DOI:** 10.1128/mra.00304-25

**Published:** 2025-05-30

**Authors:** A.C.H. Wai, G.K.K. Lai, F.C.C. Leung, S.D.J. Griffin

**Affiliations:** 1 Shuyuan Molecular Biology Laboratory, The Independent Schools Foundation Academy, Hong Kong, China; Rochester Institute of Technology, Rochester, New York, USA

**Keywords:** *Gulosibacter*, toluene, xenobiotic compounds, biodegradation

## Abstract

*Gulosibacter* spp. are known as consumers of xenobiotics and, though widely distributed in the environment, are infrequently reported. *Gulosibacter* sp. ACHW.36C was isolated from a cleaning sponge contaminated with a petroleum-based hoof oil, and its complete genome (2,748,452 bp, 67.61% G+C) established through hybrid assembly.

## ANNOUNCEMENT

The Microbacteriacean genus *Gulosibacter*, which is currently represented by only 35 isolates across eight species, was so named for its ability to feed on xenobiotics (1). Strains have been recovered from soil (2), ocean sediment (3), cows (4), and rhesus monkeys (5) and detected in cheese (6) and in a fruit-packing facility (7). In humans, *Gulosibacter* spp. are not a proven cause of infection but have nevertheless been isolated from blood samples (8) and ear infections (9). Type strain *Gulosibacter molinativorax* ON4 and *Gulosibacter* sp. YZ4 are efficient degraders respectively of molinate (a thiocarbamate herbicide) and phenol (1, 10), and here, *Gulosibacter* sp. ACHW.36C was found able to consume toluene.

Pieces of an old cleaning sponge (~2 g) used to apply petroleum-based hoof oil to horses in Hong Kong (collected: 22.515231N 114.108491E; 13 September 2019) were immersed in 8 mL of Luria broth containing 150 µL of toluene and incubated aerobically with shaking for 72 h. 250 µL of toluene was then spread onto Luria agar (11) followed by 100 µL of the culture, and, after a 24 h incubation, resultant colonies were picked and passaged to purity on Luria agar. The toluene-degrading activity of ACHW.36c was confirmed by growth in Bushnell-Haas broth (12) with toluene as the only carbon source. A single colony of the strain was streaked onto Luria agar for overnight growth before harvesting for DNA extraction (DNeasy PowerSoil Pro Kit, Qiagen GmbH, Hilden, Germany). All incubations were at 27°C.

Illumina MiSeq (v3 chemistry) and Illumina NovaSeq 6000 platforms were used to sequence paired-end short-read sequencing libraries prepared using respectively the NexteraXT DNA Library Preparation Kit and the NovaSeqX Series 10B Reagent Kit (Illumina, Inc.). A total of 5,929,194 raw read pairs were quality-filtered and trimmed using Trimmomatic v0.32 (13), producing 5,901,782 read pairs (mean length 253 bp) totaling to ~1.49 Gbp (mean coverage 543×). Long-read libraries prepared from the same extracted DNA using the Rapid Barcoding Kit SQK-RBK004 (without shearing or size selection) were sequenced via a Spot-ON Flow Cell (vR9), MinION sequencer, and MinKNOW v3.1.8 software, with base-calling by Guppy v2.1.3 high-accuracy mode (all by Oxford Nanopore). The final long-read data set totaling 396,106 reads was trimmed by Porechop v0.2.4, selecting 50,345 reads (350 Mbp) (mean length 6,952 bp, N50 9,298) to scaffold the assembly. Default parameters were used for all software, unless otherwise specified.

Illumina and MinION data sets were combined using Unicycler v0.4.8-beta (14) to yield a circular chromosome of 2,748,452 bp (67.61% G+C, mean coverage 128×; judged 99.23% complete [0% contamination] by CheckM v1.2.2 [15]—see also Table 1), which was submitted to National Center for Biotechnology Information (NCBI) PGAP v6.1 (16), with further protein BLAST using UniProt (17). Using GenomesDB (18) supplemented with recent accessions from NCBI (19), JSpeciesWS v.4.1.1 (20) found ACHW.36C closest to *Gulosibacter sediminis* strain PS01859 (GCA_030011715.1), with an average nucleotide identity of 91.44% (ANIb by BLAST+ [21]).

**TABLE 1:** The genome of *Gulosibacter* sp. ACHW.36c: summary statistics^*a*^

GenBank accession	CP097160
Replicons	1
Genome size bp	2,748,452
% G+C	67.61
Mean coverage	128×
CheckM completeness %	99.23
CheckM contamination %	0.00
CDSs (coding)	2447
Pseudogenes	41
tRNA	46
rRNA 5s-16s-23s	2-2-2

^*a*^ From NCBI PGAP 6.1 (16).

**Fig 1:**
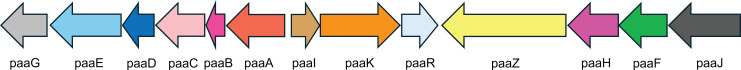
The phenylacetate (paa) gene cluster in ACHW.36c displayed according to Martin and McInerney’s 2009 study (24).

Aromatic degradation in ACHW.36c may be facilitated by a catechol 2,3-dioxygenase (EC 1.13.11.2, *hpaD*) predicted at locus M3M28_01580 (22) and/or via a thioester-based pathway suggested by the phenylacetate (paa) 13-gene cluster at M3M28_07130 to M3M28_07190 (23). The configuration of the paa cluster is shown in Fig. 1 displayed according to Martin and McInerney (24).

## Data Availability

Sequence data for *Gulosibacter* sp. ACHW.36C (CP097160) is available through NCBI under GenBank assembly GCA_023370115.1, with raw read data at SRX28113303, SRX28113302, SRX16018262 (NovaSeq); and, SRX28113305, SRX28113304, SRX16018263 (MinION).
